# Loss of Genetic Diversity Means Loss of Geological Information: The Endangered Japanese Crayfish Exhibits Remarkable Historical Footprints

**DOI:** 10.1371/journal.pone.0033986

**Published:** 2012-03-28

**Authors:** Itsuro Koizumi, Nisikawa Usio, Tadashi Kawai, Noriko Azuma, Ryuichi Masuda

**Affiliations:** 1 Creative Research Institution, Hokkaido University, Sapporo, Hokkaido, Japan; 2 Graduate School of Environmental Earth Science, Hokkaido University, Sapporo, Hokkaido, Japan; 3 Center for Transdisciplinary Research, Niigata University, Niigata, Japan; 4 Center for Toki and Ecological Restoration, Niigata University, Sado, Japan; 5 Fisheries Research Department, Wakkanai Fisheries Research Institute, Wakkanai, Hokkaido, Japan; 6 Nodai Bioresource Institute, Tokyo University of Agriculture, Abashiri, Japan; 7 Department of Natural History Sciences, Faculty of Science, Hokkaido University, Sapporo, Japan; Lund University, Sweden

## Abstract

Intra-specific genetic diversity is important not only because it influences population persistence and evolutionary potential, but also because it contains past geological, climatic and environmental information. In this paper, we show unusually clear genetic structure of the endangered Japanese crayfish that, as a sedentary species, provides many insights into lesser-known past environments in northern Japan. Over the native range, most populations consisted of unique 16S mtDNA haplotypes, resulting in significant genetic divergence (overall *F*_ST_ = 0.96). Owing to the simple and clear structure, a new graphic approach unraveled a detailed evolutionary history; regional crayfish populations were comprised of two distinct lineages that had experienced contrasting demographic processes (i.e. rapid expansion vs. slow stepwise range expansion) following differential drainage topologies and past climate events. Nuclear DNA sequences also showed deep separation between the lineages. Current ocean barriers to dispersal did not significantly affect the genetic structure of the freshwater crayfish, indicating the formation of relatively recent land bridges. This study provides one of the best examples of how phylogeographic analysis can unravel a detailed evolutionary history of a species and how this history contributes to the understanding of the past environment in the region. Ongoing local extinctions of the crayfish lead not only to loss of biodiversity but also to the loss of a significant information regarding past geological and climatic events.

## Introduction

Recent human activities have threatened considerable numbers of species and populations in nature that, in turn, have significantly affected ecosystem functioning and provision of ecosystem services [1], [2]. Genetic diversity is considered to affect population viability and evolutionary potential under changing environmental conditions [3], [4]. Thus, conserving intra-specific genetic variation has become one of the central issues in conservation biology [5].

Another important, but not fully stressed, aspect of genetic diversity is the historical footprints of past environments. Following dynamic changes in the environment, extant species have experienced various demographic events, such as range expansion, vicariance, extinction and dispersal, over the past millions of years and especially during the Quaternary Ice Age [6], [7]. Such species history may be engraved on its genes [6], [7]. Mutation rates in DNA may be sufficiently low (e.g. a few percent per million years), such that observed genetic variation may reflect the species' history over thousands or perhaps millions of years. Contemporary distributions of genotypes or haplotypes may have been generated by multiple demographic events in response to past geological and climate forces on species habitat ranges. Thus, by examining the genetic structure of species, we can infer not only species history but also past environments [8]–[11]. For example, many animals and plants in Europe possess high genetic diversity and older genotypes in southern regions (e.g. the Balkan, Italian and Iberian peninsulas) and low diversity with more recent genotypes in northern regions (e.g. Scandinavia, UK) [6], indicating post-glacial colonization from the south, consistent with geological evidence that the northern regions had been covered by ice-sheet during the glacial periods [6]. Such genetic information is particularly useful where geological processes are difficult to infer due to dynamic tectonic and volcanic activities.

Biological clues to our past environment are disappearing due to ongoing local extinctions. In addition, other anthropogenic activities, such as species introduction, transplantation or farming, have been shown to influence the original genetic structure [12], [13]. Because not all species possess regional histories in their genes (due to anthropogenic impacts, shallow population history or high gene flow [14], [15]), we need to identify geologically critical species, i.e. the species that possess valuable information on geological and climatic histories before they have been extirpated. Highly sedentary species or species whose dispersal is constrained by physical barriers may satisfy this criterion because continuous and/or long-distance dispersal which would otherwise replace the historical footprints with contemporary patterns of gene flow will not be present [14], [15]. Freshwater fishes (dispersal constrained within freshwater drainages), island species and sedentary gastropods have been shown to have simple genetic structures reflecting past environmental conditions [8]–[11].

In this paper, we clearly show that a threatened freshwater crayfish contains significant paleo-geological and climatic information in the distribution of genetic variation and that such historical footprints are in danger of disappearing due to ongoing local extinctions. Our model species was the endangered Japanese crayfish, *Cambaroides japonicus*, which is endemic to northern Japan. More detailed knowledge of past geology and climate conditions in the Japanese archipelago has been growing in the last few decades [16], but is still incomplete due to dynamic topological shifts resulting from tectonic activities (e.g. active historical volcanism). This pattern contrasts with Europe and North America which were covered by ice sheets at the last glacial maximum (LGM, ca. 20,000 years ago). Due to lack of obligate freshwater fishes in northern Japan, genetic variation in the Japanese crayfish could be highly informative for phylogeographic analysis. Because this small and slow-moving species is distributed patchily in mostly small headwater streams isolated on several islands [17], we expected that it would exhibit significant genetic structuring that might provide insights on the processes of island formation, connectivity and colonization. Through mitochondrial DNA (mtDNA) analysis, we show that the genetic structure of the crayfish throughout the species range was among the simplest and clearest ever reported for any wild animal or plant populations. This genetic structure, however, was partly inconsistent with current geology and general phylogeographic patterns and we discuss potential mechanisms for generating those inconsistencies.

## Materials and Methods

### Ethics statement

Although the Japanese crayfish has been designated an endangered species by the Ministry of Environment of Japan, there is currently no law protecting this species from handling or capture [15]. Thus, all work was legally conducted without acquiring a permit from the Hokkaido Government or the Ministry of Environment of Japan. For conservation reasons, however, we performed non-destructive sampling whenever possible.

### Biogeography of Japan

**Figure 1:**
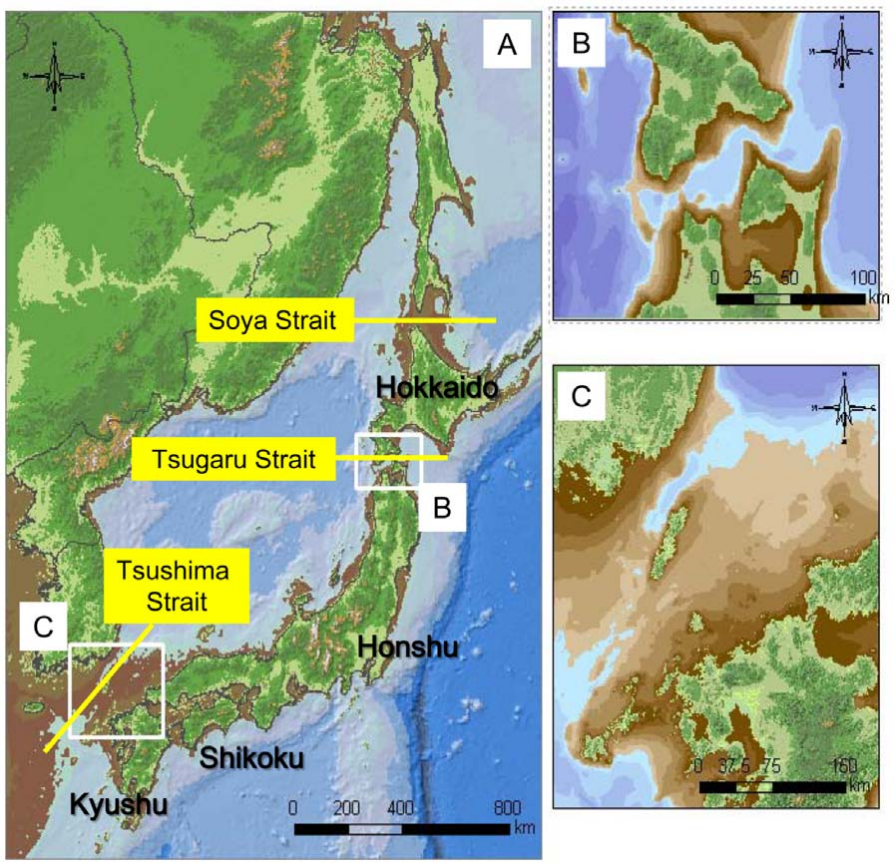
Current lands of Japan and the neighboring countries (greenish area with black contour). Presumed past land boundaries are indicated by brown-white area (white denotes 120–140 m) when sea levels decreased by 140 m during the last glacial maximum estimated from a bathymetric map (A). Top right and bottom right panels are magnified maps around the Tsugaru Strait (B) and Tsushima Strait (C), respectively, which are about the threshold levels for the existence of land bridges.

The Japanese archipelago exhibits a high endemism in fauna and flora in which speciation has occurred in isolation after colonization from the eastern Eurasian mainland [18]–[20]. Because of the lack of freshwater fishes and considerable topological changes due to volcanic activities, past environments of Japanese archipelago and the colonization patterns of animals and plants are less well-known compared to Europe and North America. Land bridges are particularly important for understanding the mode and pace of species divergence in Japan; the Tsushima Strait (130–140 m in depth), Tsugaru Strait (130–140 m in depth) and Soya Strait (50–60 m) would have been land bridges during lower sea levels and the main colonization routes to the archipelago (Figure 1). Fauna and flora in Honshu, Kyushu and Shikoku islands have probably been colonized via the Korean Peninsula through the Tsushima Strait, whereas those in Hokkaido arrived from Sakhalin Island through the Soya Strait [18]–[20]. The Tsugaru Strait has acted as a significant barrier, creating the two Japanese bioregions (i.e. Hokkaido and the other main islands).

Despite much research, there are still controversies as to if and when the Tsugaru and Tsushima straits became land bridges [18], [20]–[22]. The main challenges resolving them are that (1) landscapes have dramatically changed during the Quaternary due to tectonic and volcanic activities and (2) current ocean depths in both straits are 130–140 m, nearly equivalent to lowered ocean surface levels during the ice ages (80–140 m, [23])(Figure 1). In the past few million years, salinities of the Sea of Japan decreased several times due to closure or attenuation of these straits [24], [25], which might have caused mass extirpations or extinctions of some marine species [26].

No consensus has been reached on the locations and extents of land bridges in the Tsugaru Strait. Some biologists suggest from bio- or phylogeographical analyses that bridges in the Tsugaru Strait had emerged in the middle and/or late Pleistocene [27], [28]. Geologists, on the other hand, have reported contrasting results based on historical sea level changes, a salinity-based model or bottom core sampling. Some have suggested that land bridges existed during the LGM [29], while others have found no direct evidence of a land bridge during the past 16 million years [21], [30], [31]. Based on past mammal fauna from fossil records, Kawamura [31] proposed that there was a temporal ice bridge, not a land bridge, during the LGM.

Phylogeographic studies of mammals, insects and plants showed contrasting patterns with or without significant divergence associated with Tsugaru Strait [20], [28], [32]–[35]. These taxa have a greater variety of dispersal modes with potential to cross the strait, such as swimming, rafting, drifting or flying. Therefore, studies of freshwater species, such as fishes and amphibians, would provide more direct evidence for a marine barrier or land bridge. However, no rigorous examination has been performed so far, due to the paucity of freshwater vertebrates that inhabit both Hokkaido and Honshu, which by itself may suggest that land bridges in the Tsugaru Strait were ephemeral connections between islands. Limited data suggest that the Tsugaru Strait has been a significant barrier for a freshwater sculpin (*Cottus nozawae*) since the early Pleistocene (1.5 Myr) [36], whereas no apparent vicariance was observed in a freshwater lamprey (*Lethenteron* sp. N) [37]. These species, however, may be relicts or landlocked forms of anadromous ancestors, so they could have crossed the ocean in the past. The phylogeography of Japanese crayfish may thus resolve some of the questions regarding historical colonization routes or barriers to freshwater faunal dispersal in the Japanese archipelago.

### Japanese crayfish

Among more than 600 freshwater crayfish species in the world, most inhabit North America (59%) and Oceania (23%), followed by South America (10%) and Europe (5%)(calculated from Crandall and Buhay [38]). There are only seven species of crayfish in Asia (1.1%) and nine in Madagascar (1.4%), although other phylogenetic analyses have suggested that only four species exist in Asia [39], [40]. The Japanese crayfish is a native species endemic to northern Japan and the only one to occur in the archipelago. Its old history, remnant distribution and low species diversity make this Asian crayfish particularly important, especially because phylogenetic analyses have inferred that it is basal to other Northern Hemisphere crayfish species [39], [40].

**Figure 2:**
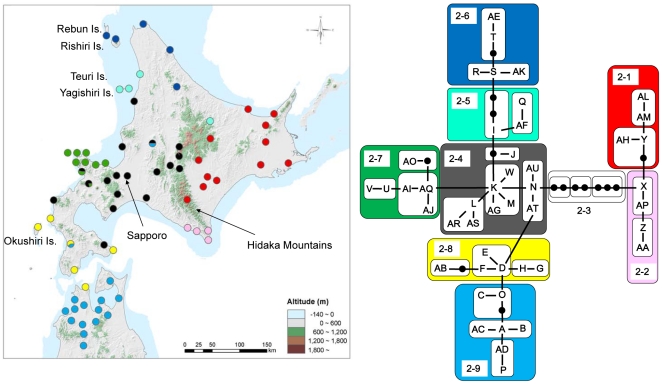
The minimum spanning network of 16S mtDNA in the Japanese crayfish (*Cambaroides japonicus*) and the distribution of level-2 nested clade (represented in the same colors). Note that the network configuration and distribution of haplotypes and nested clades are matched very well (e.g. interior haplo-groups distribute central areas, whereas tip haplo-groups distribute peripheral areas). All islands share haplotypes with the nearest main islands, including southern Hokkaido and northern Honshu. Black circles in the network represent missing haplotypes.

The Japanese crayfish is a small (<6 cm in total length), slow-moving crayfish that inhabits mostly small headwater streams [17], [41]–[43]. Terrestrial dispersal appears to be very limited due to low activity. The crayfish lack a free-swimming plankton stage [44] and long-distance dispersal via water current is presumably rare. Despite its low dispersal ability and high dependence upon freshwater environments, however, the Japanese crayfish inhabits Hokkaido and northern Honshu, as well as small islands near Hokkaido (Figure 2). Thus, the genetic structure of the crayfish may reflect paleogeological processes. Although there is no fossil or other historical record, the Japanese crayfish might have been distributed in middle or southern Honshu because the most closely related species *Cambaroides similis* inhabits the Korean peninsula. A basal lineage to the Japanese crayfish may have colonized from the Korean peninsula, which had been connected to southern Honshu during glacial periods [18]–[20].

The Japanese crayfish currently suffers from various threats to extinction: habitat loss, water pollution, timber logging, overharvest and invasion by an alien crayfish (signal crayfish, *Pacifastacus leniusculus*) are the major causes of local extinction [17], [42]. Crayfish populations have nearly disappeared from one of the largest rivers in Hokkaido (Kushiro River) in the past 30–50 years following invasion by the signal crayfish. The Japanese crayfish is considered as “vulnerable” in the Japanese Red data book [45], but no practical conservation policy or effort has yet been made.

### Sampling and DNA analysis

We collected a total of 600 crayfish from 71 locations covering the entire native range, including two main islands (Hokkaido and Honshu) and five small islands near Hokkaido (Okushiri, Rishiri, Rebun, Teuri and Yagishiri, Figure 2, Table S1). No permission was required to collect the crayfish or to enter the public lands where the samples were collected. Because preliminary analysis showed low genetic diversity within and high diversity among locations (see Results), sampling effort was directed toward collecting from a larger number of locations rather than large numbers of individuals within locations to maximize information at the species level (average 8.5 individuals per location). Genetic diversity (i.e. number of haplotypes within populations) and genetic divergence (i.e. *F*_ST_ and Nei's *D*_A_) were calculated (see below) using only 51 locations where enough samples (≧ 5 individuals) were collected (average; 11.0 individuals per location, range; 5–30 individuals). Muscle tissue was taken from a chelipod or a pereiopod and stored in ethanol at −20°C until DNA extraction.

Genomic DNA was extracted using Chelex100 (Bio-Rad, CA, USA) according to the manufacturer's instruction. A part of 16S rRNA mitochondrial DNA region was selected as a molecular marker because this appears to be the most powerful marker to identify inter- and intra-specific genetic diversity in freshwater crayfish [46]–[51]. A 490 base pair section of the 16S mtDNA was amplified by polymerase chain reaction (PCR) using primers 1471 (5′-CCTGTTTANCAAAAACAT-3′) and 1472 (5′-AGATAGAAACCAACCTGG-3′) [51]. Amplifications were carried out in a thermal cycler (Perkin-Elmer, CA, USA) in 20 µl of reaction mixture containing 50 mM KCl, 1.5 mM MgCl_2_, 10 mM Tris-HCl (pH 8.3), 0.2 mM dNTP, 0.5 µM of each primer and 0.5 units of taq DNA polymerase (TaKaRa, Tokyo, Japan) with ca. 10 ng of genomic DNA. The thermal cycling parameters were as follows: 95°C/2 min for hot start, 36 cycles of dissociation (95°C/30 s), annealing (55°C/30 s) and extension (72°C/1 min), followed by a further extension (72°C/19 min). PCR products were purified with QIAquick™ PCR Purification Kit (Qiagen, CA, USA). These products were sequenced using 3100 Genetic Analizer ABI Prism (Applied Biosystem, CA, USA) and the BigDye Terminator Cycle Sequencing kit (Applied Biosystem, CA, USA). To investigate the congruence between different genes, the more conservative 28S rRNA gene region in nuclear DNA was also amplified for 55 subsamples from 52 locations covering the entire distributional range (Table S1). These samples were amplified using the primers rD4.8a (5′-ACCTATTCTCAAACTTTAAATGG-3′) and rD7b1 (5′-GACTTCCCTTACCTACAT-3′) [52] with the following cyclic conditions: 95°C/2 min for hot start, 36 cycles of dissociation (95°C/1 min), annealing (50°C/1 min) and extension (72°C/1 min), followed by a further extension (72°C/5 min). Purification and sequencing were done in the same way as for the 16S mtDNA analysis.

The sequences were aligned using ClustalX version 1.81 [53] and BIOEDIT version 5.0.9 [54]. Due to the extremely low diversity in 28S rRNA sequences, population genetic analysis was not performed except for describing a haplotype network (i.e. minimum spanning network) using ARLEQUIN version 2.001 [55]. Therefore, the subsequent analyses were only performed using 16S mtDNA. Population differentiation was calculated as *F*_ST_ and Nei's *D*_A_, implemented in ARLEQUIN [55]. These parameters were correlated with geographic (Euclidian) distance among populations to see whether dispersal is restricted (i.e. isolation-by-distance). Significance in the correlation analysis was evaluated by a Mantel test in FSTAT [56]. A haplotype network was constructed based on the minimum spanning network using ARLEQUIN. Network loops were resolved based on the criterion of Crandall and Templeton [57] and haplotype distributions. The haplotype network was then hierarchically nested following Templeton and Sing [58]. We also tested for the effects of natural selection and/or past demographic change based on Tajima's D and mismatch distribution implemented in ARLEQUIN. If Tajima's D shows a negative value, stabilizing selection and/or rapid population expansion is suggested. A positive value, on the other hand, indicates balancing selection and/or population subdivision. A unimodal mismatch distribution, together with a negative Tajima's D, strengthens the inference of rapid population expansion. No significant deviation of Tajima's D interprets selective neutrality on mtDNA haplotypes.

Patterns of colonization can be inferred once an ancestral population has been determined [59]–[61]. Slatkin [59] mathematically formulated a stepwise colonization model derived from one proposed by David Good in his unpublished manuscript, which is qualitatively similar to the “stepping stone dispersal” model of Ponniah and Hughes [62]. In Good's model, an ancestral population gives rise to one neighbouring population with the same allele frequency, and they are subsequently isolated (i.e. no gene flow following colonization). After a time, which allows the two populations to diverge, the new population itself gives rise to another population in the same manner. That is, a new population is colonized always from the adjacent (second newest) population. Under the gradual range expansion model, genetic divergence is expected to be higher among older populations than among more recent ones even over the same geographic distances. Unlike general isolation-by-distance patterns, Slatkin [59] showed that estimated gene flow between a pair of populations (*M*), which is calculated from the inverse relationship with pairwise genetic divergence, i.e. *M* = (1/*F*_ST_−1)/4, did not depend on geographic distance between the two populations. Instead, the pairwise gene flow was *positively* correlated with the distance between the ancestral population (i.e. the original area) and one of the populations closer to the ancestral population (i.e. older population of the pair). In other words, the level of gene flow would be higher when both populations are more distant from the ancestor (i.e. more recent). In this stepwise colonization process, therefore, genetic divergence should be *negatively* correlated with the distance from the ancestral population [59], [61], [62] because of the inverse relationship between gene flow and divergence. In the present study, we plotted the distance from an ancestral population against Nei's genetic divergence *D*_A_, instead of *M* or *F*_ST_, because *M* cannot be calculated when *F*_ST_ is zero and because *F*_ST_ ranges only from zero to one. Similarly, gene diversity should be negatively correlated with distance from the ancestral population, because intra-population genetic diversity should be higher in older populations than in more recently founded ones [63].

We also developed a new graphical approach to investigate the colonization process in the Japanese crayfish. Based on coalescent theory, the relative ages of each haplotype can be compared within a haplotype network: interior haplotypes in a network should be older and tip haplotypes should be younger [57], [64]. Therefore, after determining the relative ages of each haplotype from the network, distributions of each haplotype were projected on maps in the order of their estimated ages (i.e., from the ancestral haplotype to the most recent one with one mutation step in each projection). In other words, if 10 mutation steps separated the oldest and newest haplotypes, 10 maps were generated that represent the different ages for each haplotype. By looking at the distributions of old and recent haplotypes, the direct spread of haplotypes could be visualized.

## Results

**Figure 3:**
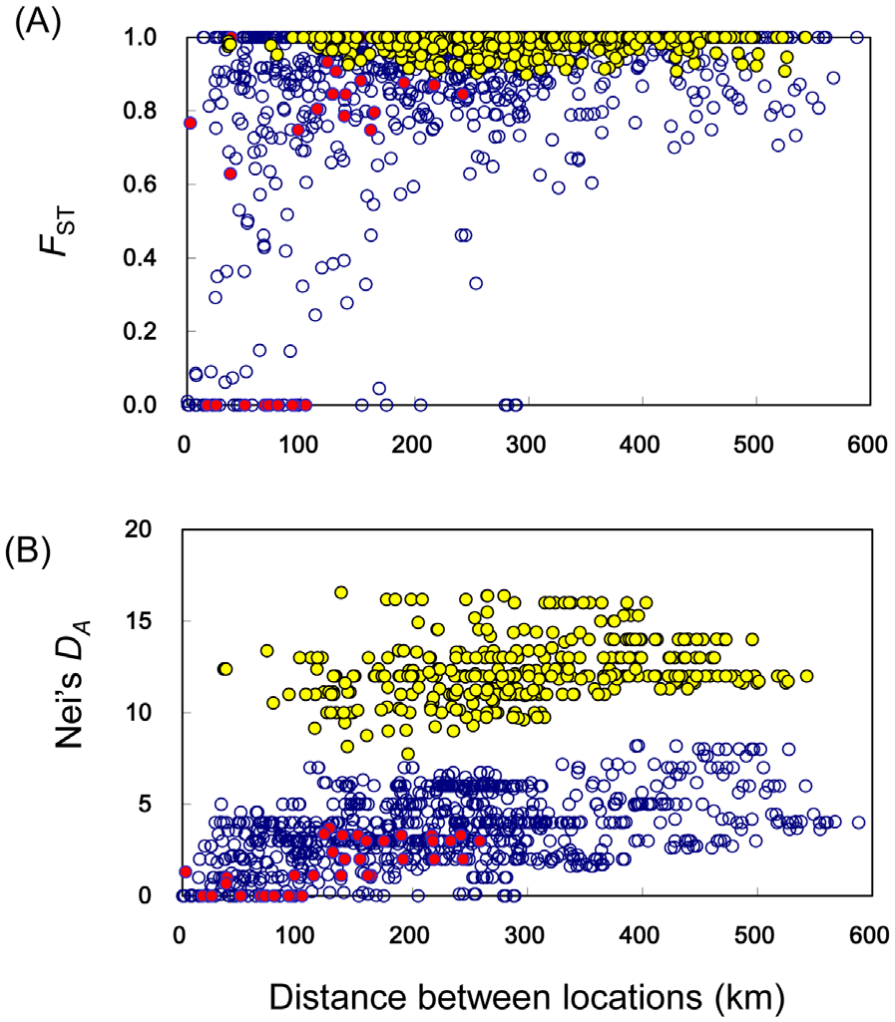
Correlation between geographic distance and genetic distance (upper panel A for *F*_ST_, lower panel B for *D*_A_) for tests of isolation-by-distance in the Japanese crayfish. Open, red and yellow circles represent comparisons within the western lineage, within the eastern lineage and between the lineages, respectively. *F*_ST_ and Nei's *D*_A_ within western lineage, within eastern lineage and between the lineages all showed significant positive correlations with geographic distance (see text).

Based on a 490 bp of 16S mtDNA, 46 haplotypes were detected among 600 individuals from 71 locations (Figure 2, Table S1). All the nucleotide sequences are available from GenBank/DDBJ/EMBL databases (accession numbers AB508237–AB508282). Genetic diversity was extremely high among and low within locations. Of the 46 haplotypes, 28 (61%) were unique to single locations and 38 (83%) were unique to single regions (a region is a set of locations in close proximity, <ca. 50 km). Only eight haplotypes (17%; haplotypes C, D, I, K, N, O and Y) were shared among different regions (Figure 2, Table S1). Of 51 locations where five or more samples were collected (average; 11.0 individuals per location), 35 localities (69%) possessed just single haplotypes. The high levels of monomorphism within locations are not likely the result from sampling bias associated with relatively small sample sizes (i.e. 11.0 individuals on average) because 61 individuals from four adjacent locations (2–24 km apart) in a single river (the Sorachi River) contained only a single haplotype and 74 individuals from four neighboring rivers (0.3–8 km apart) in the Shakotan region shared the same haplotype, except for one individual. We pooled the Sorachi and Shakotan samples as single populations for generating mismatch distributions, as these analyses tend to suffer from unequal sample sizes. Because of the high diversity among and low diversity within populations, genetic differentiation was extremely high (mean pairwise *F*_ST_ = 0.96 for the 51 locations; Figure 3A). Two highly divergent lineages were observed (the eastern and western lineages, see below) and we calculated *F*_ST_ values for each lineage separately. Again, populations were highly diverged (mean pairwise *F*_ST_ = 0.87 in the western lineage encompassing 42 populations, mean pairwise *F*_ST_ = 0.65 in the eastern lineage encompassing nine populations). Weak, but significant, positive correlations were observed between pairwise *F*_ST_ and geographic distance (*r* ^2^ = 0.117, *P*<0.0001, Mantel test, Figure 3A) and between Nei's *D*_A_ and geographic distance (*r* ^2^ = 0.168, *P*<0.0001, Figure 3B), suggesting that dispersal is restricted among neighboring locations. These relationships were qualitatively the same even when each lineage was analyzed separately (western lineage: *r* ^2^ = 0.095, *P*<0.0001 for *F*_ST_, *r* ^2^ = 0.193, *P*<0.0001 for *D*_A_, eastern lineage: *r* ^2^ = 0.425, *P*<0.0001 for *F*_ST_, *r* ^2^ = 0.127, *P* = 0.0304 for *D*_A_).

The minimum spanning network showed these two divergent lineages were separated by a minimum of seven mutation steps, with only a few missing haplotypes within each lineage (Figure 2). Mean sequence divergences were 3% and 0.5–1% between and within the lineages, respectively. The deep divergence between the lineages was not associated with Tsugaru Strait, but with mountains in Hokkaido. The lineage that includes haplotype X was exclusively found in eastern Hokkaido and is bounded to the west by the Hidaka Mountains (hereafter called the “eastern lineage”). A single haplotype (Y) dominated this area (70% of the locations: Table S1), suggesting rapid range expansion. Haplotype X was inferred as the ancestral extant haplotype in the eastern lineage due to its interior position in the network (Figure 2). It was located on the southern edge of the range for the eastern lineage. The other lineage (hereafter called the “western lineage”) was distributed across the remainder of the geographic range, spreading 600 km in a latitudinal gradient. Of 37 haplotypes in the western lineage, only five haplotypes were relatively abundant (more than 20 individuals) and found in multiple locations (haplotypes C, D, K, I and S: Table S1). Haplotype K was strongly inferred as the ancestral or oldest extant haplotype in this lineage due to a high relative abundance and its central position in the network. Also, haplotype K appeared around the current geographic center of the crayfish distribution (near Sapporo, see Figures 2 and 5), indicating a refuge in this area. Tajima's D for all data and for the two lineages separately showed no evidence for natural selection, historical bottleneck or demographic expansion events.

Geographic distributions of haplotypes and nested haplotype clades were concordant with the haplotype network (Figure 2). Haplotypes separated by single mutations were almost always nearest neighbors, consistent with the positive correlation of genetic and geographic distances (Figure 3). The clustering patterns, however, did not fully conform with current geography in terms of ocean barriers. Despite a few sharing haplotypes among different locations within the main islands (i.e., Hokkaido and Honshu), all small islands had the same haplotypes as their nearest sampling location on Hokkaido, except for two unique haplotypes (Q and AF) found on Yagishiri Island. In addition, haplotype F was found in the southern tip of Hokkaido and northern tip of Honshu (Figure 2, Table S1), suggesting no prominent vicariance influence by the Tsugaru Strait.

**Figure 4:**
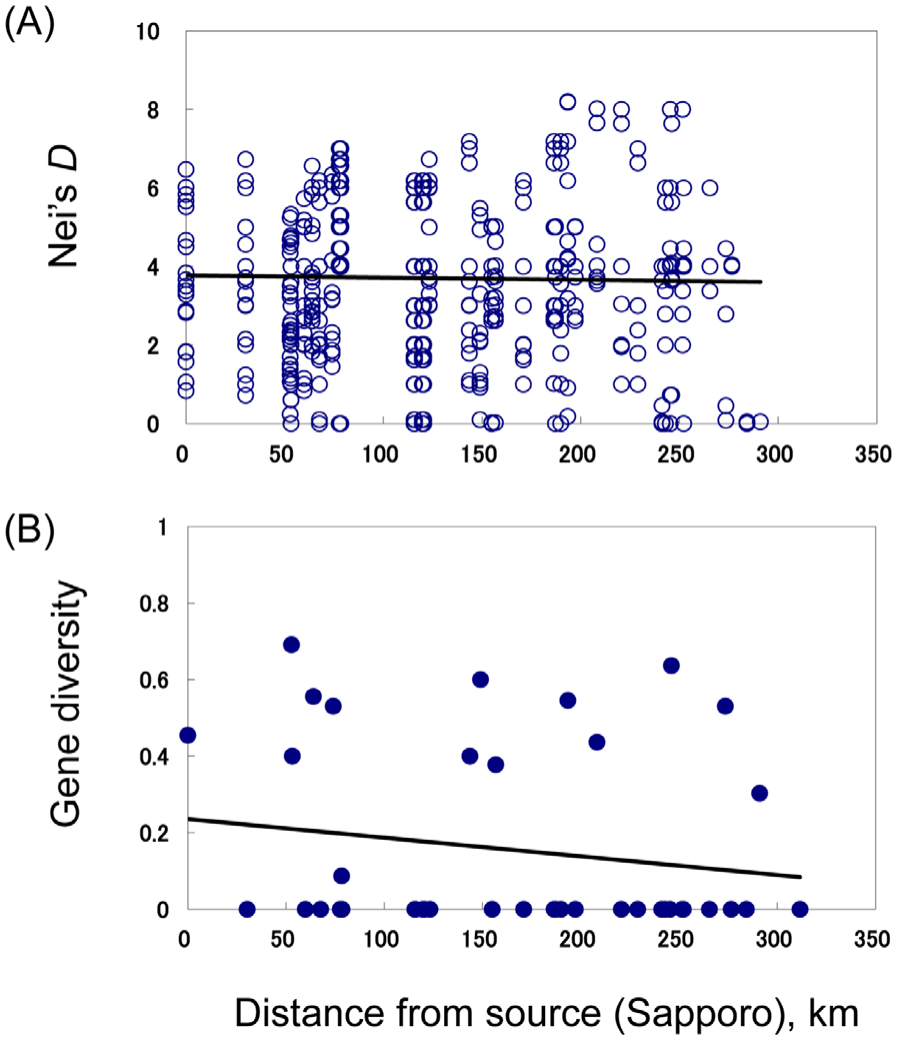
Tests of sequential colonization processes in the western lineage of the Japanese crayfish. Despite that negative correlations are expected under the Good's model of colonization, no correlation was evident between: A) Nei's genetic distance *D*_A_ and geographic distance from the ancestral population (*r* ^2^ = −0.0004, Mantel's *P*>0.05), and B) gene diversity and geographic distance from the ancestral population (*r* ^2^ = −0.029, Pearson's *P*>0.05).

We tested the Good's stepwise colonization model [59], using the oldest inferred haplotype K, assuming the Bankei sample from the Sapporo area as the ancestral location for distance calculations. Contrary to expectations, neither the genetic divergence (*D*_A_; *r* ^2^ = −0.0004, Mantel's *P*>0.05) nor the intra-population genetic diversity (*r* ^2^ = −0.029, Pearson's *P*>0.05) was correlated with geographic distance from the ancestral area (Figure 4).

**Figure 5:**
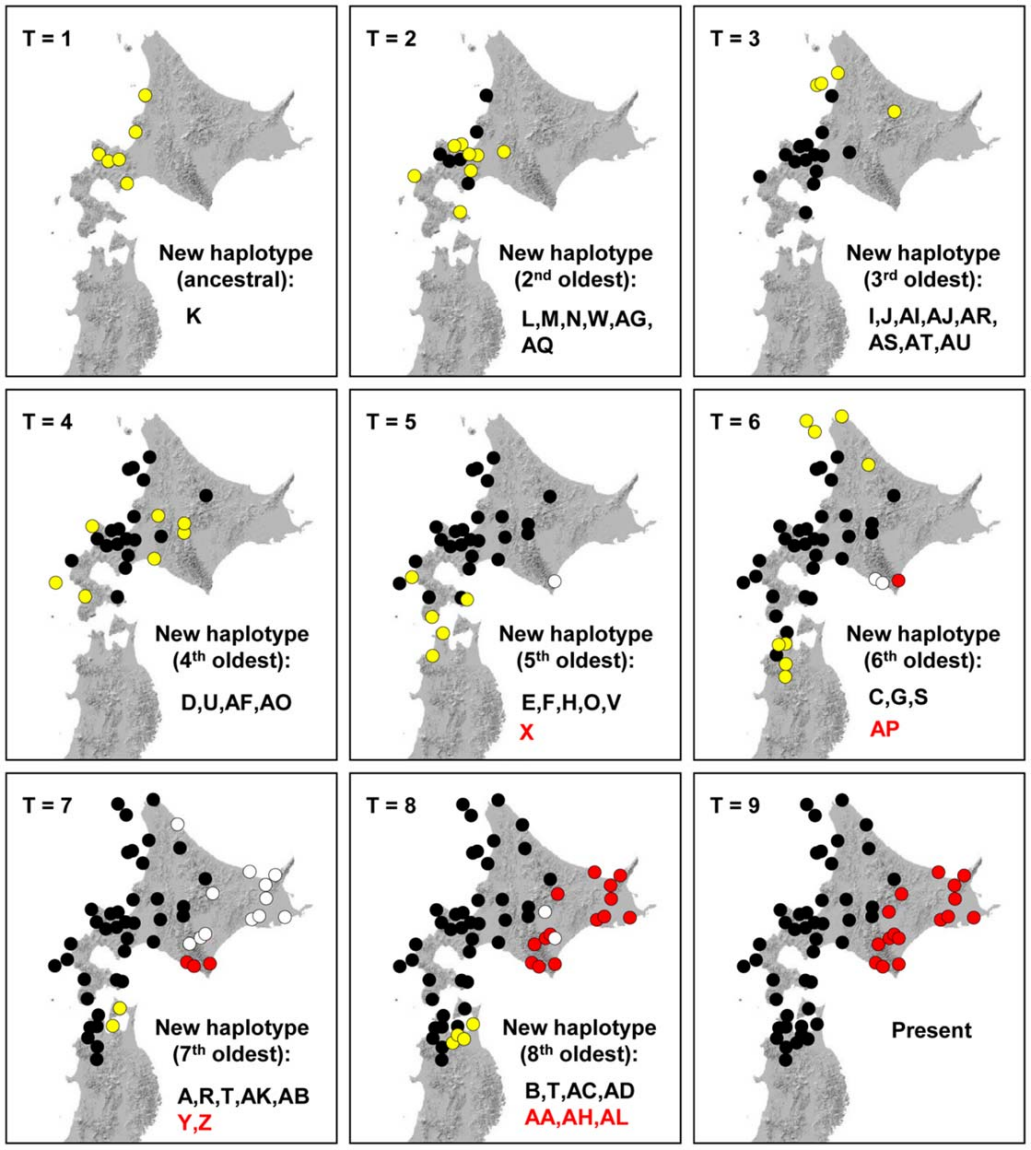
Genealogical spread of the haplotypes of the Japanese crayfish. Each map represents a certain time period according to a single mutational step (T = 1 when the most ancestral haplotype K appeared). Yellow and white circles are newly emerged haplotypes in the western and eastern lineages, respectively, in each period. Black and red circles represent the western and eastern lineages, respectively. The Japanese crayfish crossed the Tsugaru Strait between T4 and T5. Subsequently, the haplotype Y rapidly spread over the eastern Hokkaido between T6 and T7.

Our new projection method, however, showed a different colonization pattern (Figure 5). We plotted the distributions of haplotypes of the same age from the ancestral haplotypes K and X for the western and eastern lineages, respectively, in increments of single mutational steps. To standardize the time periods of the western and eastern lineages, the most recent (tip) haplotypes in each lineage (A and AL, respectively, see Figure 2) were set to the same time period (i.e. most recent). This reverse coalescent plot (Figure 5) clearly demonstrated that derivative haplotypes from ancestral populations at the geographic center colonized to the north and south at a roughly equivalent pace in the western lineage, whereas the eastern population experienced a period of rapid expansion relatively recently.

Based on a 453 bp of 28S nuclear DNA, only two haplotypes were detected from 52 populations comprising 55 individuals (Figure S1, Table S1). Both nucleotide sequences are available from GenBank/DDBJ/EMBL databases (accession numbers AB669198 and AB669199). The two sampled haplotypes, however, were highly diverged, separated by eight mutation steps and three deletions/insertions (Figure S1). One haplotype was exclusively found in the eastern lineage identified from the mtDNA analysis and the other in the western lineage. This result perfectly matches with other phylogeographic inferences from mtDNA variation.

## Discussion

### Genetic structure of the Japanese crayfish

Japanese crayfish showed one of the highest levels of genetic differentiation (*F*_ST_ = 0.96) reported for any organism [65]. The average nucleotide divergence (3.0% between the two lineages, 0.5–1.0% within lineages) in the 16S mtDNA region likewise infers extreme isolation of these lineages and individual populations, considering that this region evolves slowly and is generally used as an inter-specific and intra-specific genetic marker [66]. Other crayfish species showed similar levels of divergence at the 16S region at equivalent geographic scales in North America (up to 4.2%, [48]), Europe (0.3–2.0%, [49]) and Australia (1.8–3.4%, [50]). Applying a molecular calibration for decapod crustaceans 16S mtDNA (0.65–0.88% pairwise sequence divergence per Myr, [66]), the divergence between the two Japanese lineages falls in the Pliocene (3–5 Myr) and within the Pleistocene (<1 Myr) for each lineage. Although the molecular clock calibration may only be approximate [67], our preliminary estimate indicated that the extant genetic diversity has been generated over a few million years. Thus, the clear genetic structure has been preserved even under the climatic and geological oscillations in multiple glacial cycles.

Because of prominent divergence in both mtDNA (16S rRNA) and nuclear DNA (28S rRNA) and of geographically separated distributions, the western and eastern lineages may possibly represent two cryptic species or sub-species. In other countries, some crayfish lineages that showed equivalent divergence to the western and eastern Japanese crayfish lineages presented here were treated as separate species or sub-species [46], [49]. However, the morphological characters of the western and eastern lineages are very similar [68] and they can reproduce viable offspring (T. Kawai, unpublished data). Therefore, we considered the western and eastern lineages as intra-specific variation in this paper. Further taxonomic studies are needed to verify their phylogenetic relationship.

The pronounced genetic structure of the Japanese crayfish is likely due to its patchy distribution, low dispersal ability and small effective population sizes. High levels of genetic differentiation at each locality (e.g. river, tributary) indicated highly localized populations with almost no gene flow with other river drainages (at least in the western lineage). The crayfish can walk on the land, but such movements may rarely contribute to gene flow to other river systems. Colonization or gene flow to other drainages may be achieved by terrestrial dispersal on extremely rare occasions or, more likely, through drainage rearrangements, such as stream capture or drainage diversion [69], [70]. Low genetic diversity within populations strongly indicates small effective population sizes, probably accompanied by founder effects or bottlenecks. A founder effect is very likely, given the low dispersal ability of the species. Demographic population sizes may be also small because distributions are generally restricted to small headwater streams [17].

The Japanese crayfish represents a paradox with regard to genetic history. Extinction of local populations could naturally occur due to biological characteristics such as small population size and low dispersal ability. However, patterns of genetic variation have been maintained for a long time, possibly several million years. Supporting evidence for the stability of genetic structure is the low frequencies of missing haplotypes within lineages. This is surprising, considering that each local population may be ephemeral and has a unique haplotype. There may be some mechanisms for long-term persistence even in small populations [71]. Alternatively, even though each local population is ephemeral, extinction-colonization dynamics may maintain long-term genetic variation at metapopulation levels [72].

### Footprints of the past environment

The evolutionary history of the Japanese crayfish provided many insights into the past geography and climate in northern Japan. The only significant barrier to the crayfish was the Hidaka Mountains, separating the western and eastern lineages. Surprisingly, current ocean barriers have not significantly affected the genetic structure of the crayfish. All small islands shared the haplotypes with the nearest areas of Hokkaido Island, indicating land connections existed in the recent past, possibly in the LGM (ca. 20,000 years ago). Although crayfish had once been distributed to different areas as a traditional medicine 200 years ago [73], human transportation is unlikely to explain the current genetic structure. If translocations were frequent and widespread, the genetic structure of the crayfish would not have been evident over the native range due to genetic admixture. Also, the existence of unique haplotypes in one island (Yagishiri) indicates that the island population is native. Nevertheless, we cannot fully exclude the rare opportunity of human transportation among nearby regions: for example, a land bridge between Hokkaido and Okushiri Islands (>400 m in ocean depth) seems unrealistic. Another potential way for crayfish to cross the ocean would be dispersal during periods when salinity of the Sea of Japan had decreased. This seems unlikely, however, because this pattern is not evident in more mobile freshwater fishes that would have crossed the strait.

Our results suggest the existence of a land bridge connection across the Tsugaru Strait. Genetic divergence was not pronounced among the crayfish populations in southern Hokkaido and northern Honshu. In addition, the closest populations in Hokkaido and Honshu, in terms of linear geographic distance, shared the same common haplotype. Because there is no strictly freshwater fish species distributed both in Hokkaido and Honshu (although a few landlocked fishes exist), the genetic structure of Japanese crayfish provides the first strong biological indication of the existence of a land bridge in the Tsugaru Strait. Dating of a putative land bridge has been controversial, but if we use the Schubart *et al.*'s [66] mutation rate estimates for decapods (0.65–0.88% pairwise sequence divergence per Myr), the land bridge might have existed approximately 0.9–1.3 million years ago, considering crayfish had undergone four mutational steps (i.e. 0.82% substitutions) after the colonization of Honshu Island (Figure 5). Again, caution is needed to estimate the dating, but our divergence time agrees with a previous study on landlocked sculpin in northern Japan isolated by the Tsugaru Strait since approximately 1.5 million years ago [36]. The existence of multiple land bridges in different periods is also possible, given that the same haplotype is shared between southern Hokkaido and northern Honshu. In that case, a land bridge(s) could have been existed much more recently than 0.9–1.3 million years ago. More polymorphic, fast-evolving, molecular markers, such as mtDNA COI (cytochrome oxidase subunit I), should provide finer resolution for dating divergence dates, including those for the small islands near Hokkaido.

The spread of a single haplotype (Y) over eastern Hokkaido may be due to rapid habitat shifts during the ice ages. Eastern Hokkaido is characterized by flat topology and colder temperature with large areas of wetlands compared to other parts of northern Japan. Yonekura *et al.* [16] suggested that eastern Hokkaido had belonged to a discontinuous permafrost zone during the ice ages, which might have led to a widespread loss of habitat. Subsequent postglacial warming may have allowed the crayfish to colonize readily in these large, low gradient systems. Flat topology might have helped the dispersal of this sedentary species by decreasing the costs of terrestrial movements or increasing the frequency of drainage connections due to flooding. Using a molecular clock for decapod 16S with two mutational steps (0.41% substitutions) from the tip haplotype AL to the widespread haplotype Y, emergence of the latter may date back to 460 000–630 000 years ago (middle Pleistocene). This tentative estimate suggests that rapid expansion had taken place after an extinction event during one of the coldest glacial periods (i.e. 430 000 or 630 000 years ago, or Isotope Stage 12 or 16). While this calculation is based on only a few mutational steps and could have potential errors, the estimated time seems to correspond nicely to known geological events. Landlocked lamprey (*Lethenteron* sp. N) and sculpin (*Cottus nozawae*) populations displayed unique haplotypes in eastern Hokkaido [36], [37] and further research is needed to test the hypothesis of range-wide extinction and colonization events in freshwater species during the middle Pleistocene.

The genetic structure observed in Japanese crayfish may add new phylogeographic perspectives on highly sedentary species. In the Northern Hemisphere, temperate species generally have glacial refuges in the southern range of the distribution, whereas arctic species have interglacial refuges in northern range [74]. The Japanese crayfish, however, had refuges in the geographic center of the western lineage, as well as a normal southern refuge in the eastern lineage. The survival in the intermediate latitude may be due to the extremely low dispersal ability of the crayfish; they could not have tracked climate shifts during the ice ages. For example, in the severe glacial/inter-glacial cycles, southern populations might have gone extinct in a warmer period, whereas northern populations might have been extirpated in the subsequent colder period, resulting in remnant populations in the central area. The generality of the pattern could be assessed by examining the genetic structure of equally highly sedentary species.

Another unexpected pattern is the equivalent pace of the northward and southward colonization, regardless of geography and climate. We hypothesize that colonization events might have occurred along coastlines. When sea levels decreased in the glacial periods, neighboring rivers might have been connected or closer to each other downstream [75], allowing crayfish to colonize to the adjoining rivers (in north and south directions). In the newly colonized populations, new mutations were probably fixed during the subsequent isolation period (i.e. inter-glacial period). If these colonization processes were repeated during each glacial/inter-glacial cycle, we would find a genealogical pattern found in the present study. The likelihood of the inter-river colonization should not substantially differ between the north and south in the coastal dispersal scenario, compared with the colonization by dispersal across the land, which should be affected by different factors.

### Discordance with coalescent theory

Although the stepping-stone colonization process of Japanese crayfish appears to be simple (Figure 5), the observed patterns did not follow the predictions from the theory: the genetic divergence and genetic diversity were not higher among older than among younger populations (Figure 4). The results imply that mutations occurred and been fixed only in newly colonized populations, which created the distinct pattern of incremental spread. Few mutant haplotypes were found in older source populations, suggesting that mutations had rarely occurred or had rarely been fixed after the settlements of dominant haplotypes. Interestingly, cave crayfishes in the southeastern United States seem to have undergone similar stepping-stone colonization processes, although there are some differences such as moderate genetic diversity in both young and old populations [46].

The reason(s) that mutations were fixed only in newly established populations is unclear, but may be explained by either or both of the following two reasons. First, mutations may be more likely to occur in newly established populations if new habitats are less suitable (more stressful) and mutations occur in response to harsher conditions (i.e. adaptive mutation, [76]). Second, individuals with older haplotypes might have higher fitness in older habitats maintained by natural selection. However, both potential mechanisms assume adaptive mutation and natural selection on mtDNA that are not widely recognized in natural populations, although the evidence of the latter is growing [77], [78].

A more plausible explanation is the effects of population size on genetic drift. New mutations are more likely to be fixed in recently established, smaller populations due to stronger genetic drift. Under the nearly neutral theory [79], where most mutations are only slightly deleterious, genetic drift is stronger in smaller populations (younger population in this case) because larger populations could eliminate even such weaker deleterious mutations. Once populations are established, intrusions of other haplotypes, either by rare mutations or rare dispersal events, should be uncommon due to the effect of “prior residence” or “mutation surfing” [80], [81]. Spatially explicit coalescent-based simulations may reveal the biological and environmental conditions under which such stepwise, leading-edge colonization patterns are generated [82].

### Conservation implications

Biogeographic studies provide significant insights into geological and climatic processes, as suggested in the continental drift theory by Alfred Wegener [83]. With the aid of recent advances in molecular techniques and statistical tools, phylogeographic studies can reveal more details about past geological and climatic processes. To provide biological evidence for the continental drift theory, Wegener recommended studying the distributions of sedentary species, such as earthworm, land snails and/or freshwater crustaceans (e.g. 105–106 pages in [83]). Such sedentary species may also possess prominent historical footprints in their genes, as shown by the present study.

Freshwater fish have been model organisms in phylogeographic studies because their dispersal is constrained within watercourses [9], [11]. Some freshwater invertebrates, however, may be even more appropriate. Some fish populations are impacted by human activities such as exploitation and aquaculture, so the original genetic structure may not be retained due to translocations and associated loss of genetic diversity following population reduction. Unaffected freshwater invertebrates may possess significant historical information in the past, especially those inhabit less-affected headwater streams, such as planarians [84] and shrimp [85]. Recently, freshwater crayfish have been paid much attention as model species in phylogeographic studies and have contributed to the understanding of regional paleo-environment [47], [48], [62], [75].

Species with highly restricted dispersal tend to show patchy distributions and small population sizes, characteristics that make them highly vulnerable to human impacts. Local extinction of such species does not only lead to loss of local species diversity, but also to loss of the genetic legacy created over thousands of years. As a geographically critical species, conservation measures for the Japanese crayfish are urgently needed. At the least, genetic samples from as many streams as possible should be collected for future analyses in support of conservation efforts. Translocation or re-introduction should be avoided to maintain its highly unique patterns of intra-specific genetic diversity that serves as a footprint of past environmental events.

## Supporting Information

Figure S1 **The minimum spanning network of 28S nuclear DNA and the distributions of the Japanese crayfish (** ***Cambaroides japonicus*** **).**
(TIF)

Table S1 **Information on sampling location, sample size and haplotype frequency in the Japanese crayfish (** ***Cambaroides japonicus*** **).**
(TIF)
