# Supplementary figures and images for: Loss of Genetic Diversity Means Loss of Geological Information: The Endangered Japanese Crayfish Exhibits Remarkable Historical Footprints
Source: PLoS One. 2012 Mar 28;7(3):e33986. doi: 10.1371/journal.pone.0033986 (PMC3314697; doi:10.1371/journal.pone.0033986)

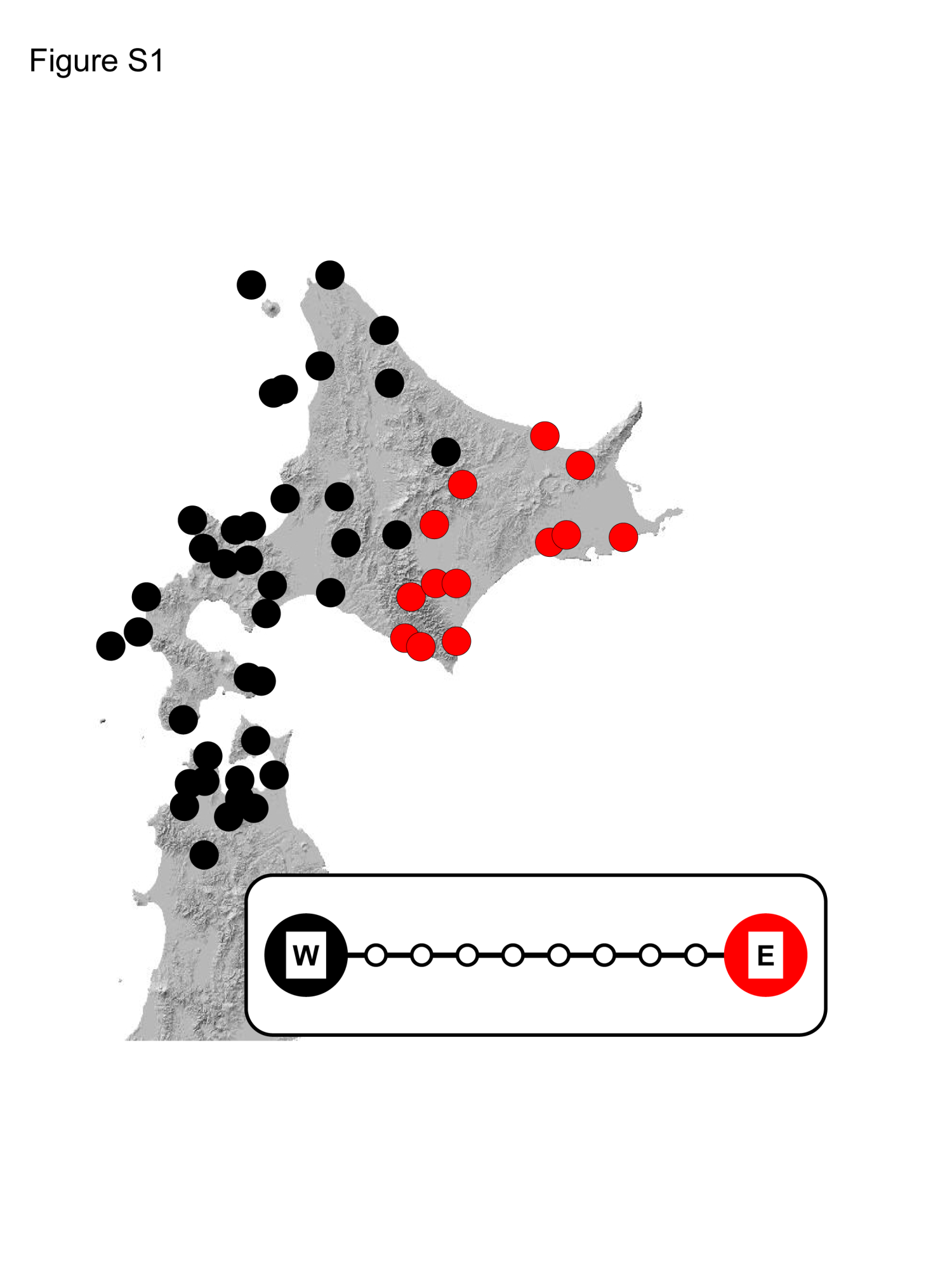

Supplement: Figure S1 — The minimum spanning network of 28S nuclear DNA and the distributions of the Japanese crayfish ( Cambaroides japonicus ). (TIF) [file pone.0033986.s001.tif]
